# Context-Dependent Effects of Maternal Behaviour on Lamb Growth in Tibetan Sheep

**DOI:** 10.3390/ani16091386

**Published:** 2026-05-01

**Authors:** Zihao Gu, Mingdi Wang, Zhong Liang, Yonggui Ma, Yinglian Qi, Jiapeng Qu

**Affiliations:** 1 School of Life Science, Qinghai Normal University, Xining 810008, China; 2 Qinghai Province Key Laboratory of Animal Ecological Genomics, Northwest Institute of Plateau Biology, Chinese Academy of Sciences, Xining 810008, China

**Keywords:** animal personality, maternal effects, Tibetan sheep, offspring growth, behavioural traits

## Abstract

Consistent behavioural differences among individuals have been documented across many animal taxa, yet their developmental consequences in domestic production systems remain less clear. This gap is especially relevant in managed environments, where food availability and ecological constraints differ markedly from those in the wild. In this study, we assessed behavioural traits in 25 Tibetan sheep (*Ovis aries*) ewes and examined their associations with early growth in their lambs under semi-captive conditions on the Qinghai–Tibetan Plateau. We quantified docility, exploration, vocal responses, and several physiological measures of responsiveness in the ewes and related these variables to lamb birth weight, early weight gain, and a composite growth index. We found that maternal docility had no significant effect on lamb birth weight, whereas maternal activity, defined as locomotor movement recorded during the open-field phase, was negatively associated with offspring birth weight. One possible interpretation is that ewes showing greater locomotor activity during the open-field phase allocate energy differently during gestation, which could limit foetal growth, although this mechanism was not directly tested in this study. These results suggest that the behavioural effects on offspring development depend strongly on the production context. In this semi-captive system, greater maternal responsiveness was not associated with improved offspring performance, unlike patterns that are often observed in wild populations. This context dependence may be relevant when behavioural traits are considered in Tibetan sheep management or breeding.

## Simple Summary

Individual behavioural differences may affect both productivity and welfare in livestock, but the evidence from domestic grazing systems remains limited. In this study, we examined whether maternal behavioural variation in Tibetan sheep was associated with lamb growth under semi-captive conditions on the Qinghai–Tibetan Plateau. Lambs born to ewes showing greater locomotor activity and stronger vocal responses tended to have a lower birth weight, whereas maternal docility showed no clear association. This pattern differs from many reports in wild animals, where higher activity or boldness is often linked to improved resource acquisition. Our results indicate that under managed feeding conditions, the developmental consequences of maternal behavioural variation may differ from those observed in wild systems and may therefore deserve consideration in Tibetan sheep management.

## 1. Introduction

Consistent among-individual differences in behaviour, commonly referred to as animal personality, are widely recognised as an important source of variation within animal populations. Such differences are often stable over time and across contexts and have been documented across a broad range of taxa, including invertebrates, fishes, birds, and mammals [1,2,3]. These behavioural differences are not merely descriptive; they have been linked to key ecological and life-history processes, including dispersal, habitat use, social interactions, and reproductive success [4,5]. Personality variation is therefore of interest not only as a behavioural phenomenon, but also because its effects on fitness-related traits may differ across ecological settings.

Beyond their ecological relevance, personality traits are increasingly being studied from a developmental perspective. Rather than being fixed properties, behavioural differences often emerge early in life and may be shaped by both prenatal and postnatal environments [6,7]. Early-life conditions can influence both the expression and temporal stability of behavioural traits, indicating that individual behavioural differences emerge through development instead of being fixed from the outset [8]. Beyond direct genetic inheritance, parental behaviour is a critical but insufficiently explained link between parental phenotype and offspring performance.

Within this framework, parental effects are a key pathway through which variations in behaviour may arise. Parental effects refer to influences of the parental phenotype or environment on the offspring phenotype that are independent of direct genetic inheritance [9,10]. These effects can comprise multiple factors, including the maternal hormonal environment, nutritional provision, and early-life social interactions [11,12]. Parental behavioural phenotypes may therefore affect their offspring’s development through behavioural interactions, prenatal conditions, or other physiological pathways [13].

In domesticated mammals, sheep (*Ovis aries*) provide a useful system for examining the links between parental phenotype and behaviour and offspring performance. Maternal behaviour in sheep has been well-studied, particularly around parturition, and it includes behaviours such as grooming, nursing, and offspring recognition, all of which are critical for early survival and development [14,15]. Moreover, variation among ewes in these behaviours has been associated with differences in lamb survival and early growth under different production systems [16,17]. Despite extensive research on maternal care and temperament in sheep, these components have rarely been examined within an integrated framework that simultaneously considers maternal personality, offspring behavioural variation, and growth outcomes. As a result, it remains unclear whether personality-related maternal effects extend beyond classical maternal care and whether they operate under livestock-specific ecological constraints.

Sheep also exhibit consistent behavioural variation that is often described in terms of temperament or personality-related traits, such as activity, fearfulness, and stress responsiveness [18]. Some of these traits show moderate repeatability and have been linked to production-related outcomes, suggesting that behavioural variation may be relevant for management and breeding. However, most existing studies have examined maternal behaviour, behavioural traits, or offspring performance separately, rather than within an integrated framework.

Consequently, it remains unclear whether variation in maternal behavioural traits is associated with offspring growth and development beyond the direct effects of maternal care, particularly under livestock production conditions in which resource availability and environmental constraints differ from those of wild systems. In addition, the extent to which offspring behavioural traits are associated with growth remains poorly understood in sheep.

Although maternal behaviour, offspring behavioural variation, and growth performance have each been examined in previous studies, these components have rarely been integrated within a unified framework under livestock production conditions. This gap is particularly relevant in Tibetan sheep, in which maternal and offspring behavioural variation may influence early development under the ecological and management constraints of the Qinghai–Tibetan Plateau. The purpose of this study was to examine whether maternal and offspring behavioural traits are associated with early growth in Tibetan sheep under semi-captive conditions on the Qinghai–Tibetan Plateau. From a scientific perspective, this study aimed to determine whether behavioural and maternal effects on offspring growth, which have been discussed mainly in wild or non-domestic systems, can also be detected in a managed livestock context. From a practical perspective, the study aimed to assess whether such behavioural variation may have potential relevance for Tibetan sheep management and breeding under plateau production conditions. Accordingly, the present study addressed two specific questions: (i) whether maternal behavioural traits are associated with variation in offspring growth, and (ii) whether offspring behavioural traits are associated with individual differences in growth and development.

## 2. Materials and Methods

### 2.1. Study Site and Population

This study was conducted at the Haibei Demonstration Zone of Plateau Modern Ecological Animal Husbandry Science and Technology, Xining, China (36°55′ N, 100°57′ E). Tibetan sheep were managed under a semi-captive production system that was typical of the Qinghai–Tibetan Plateau. Following lambing, lambs stayed with their dams until weaning. The flock was herded to graze on alpine meadow pastures during the day (approximately 06:00–17:00) and returned to the pens at dusk. While housed, the sheep had ad libitum access to drinking water and a commercially formulated concentrate supplement (Menyuan Yongxing Ecological Agriculture and Animal Husbandry Development Co., Ltd., Menyuan County, Haiyan, China). The concentrate, which was composed primarily of cereal- and oilseed-derived ingredients (such as corn, bran, and soybean meal), contained approximately 16% crude protein, 3% crude fat, and 8% crude fibre. A total of 80 ewes and 73 lambs were included in the study. The focal animals were selected on the basis of their health status. The ewes had a mean age of 2 years and a mean body mass of 44.24 (31–53.6) kg at the beginning of the study. The animals had not received prior formal training in relation to the test apparatus before behavioural testing.

### 2.2. Ethical Considerations

All experimental procedures were conducted in accordance with established animal welfare standards, with a focus on minimising stress and preventing injury to the animals. Behavioural assessments were carried out using gentle herding techniques and short observation periods, avoiding prolonged isolation or invasive manipulation. Physiological measurements were taken using brief and mild restraint, performed exclusively by trained and experienced personnel to ensure the safety and welfare of the animals throughout the procedure. The testing arena and associated experimental setup were designed to allow sufficient space for controlled movement during the behavioural assays and to avoid crowding or unnecessary physical restriction.

Animal housing, feeding, handling, and all experimental manipulations were carried out in compliance with the guidelines set by the Animal Care and Use Committee and were reviewed and approved by the Ethics Committee of the Northwest Institute of Plateau Biology, Chinese Academy of Sciences (approval number: NWIPB20170114).

### 2.3. Experimental Design and Measurement Timeline

To characterise consistent among-individual variations in behaviour (animal personality) and its physiological correlates, behavioural assays and physiological measurements were repeated across multiple developmental stages for the same individuals. In accordance with standard practices in personality research, personality was conceptualised as repeatable individual variation in behavioural responses expressed over time and, when applicable, across test situations [2,19,20]. The general testing framework used here was also informed by our previous study in Tibetan sheep [21].

The behavioural dimensions targeted in this study were (i) boldness/shyness, reflecting the tendency to take risks under exposure; (ii) exploration, quantified by locomotor activity and space use in a novel or open arena; (iii) response to novelty, indexed by the latency to approach or make first contact with an unfamiliar object; and (iv) docility/temperament, representing behavioural calmness and ease of handling, including the propensity to settle in a designated resting area after testing [2,19,21].

All behavioural tests were conducted in a purpose-built arena consisting of a series of connected compartments separated by gates. This design allowed each focal animal to pass through the assay sequence in a fixed order while avoiding direct interaction with conspecifics. Each ewe was tested individually under standardised conditions, and all behavioural responses were recorded by video for subsequent scoring. To maintain strict standardisation across individuals and sessions, the maximum duration in each compartment was predefined. In this protocol, individuals completed an open-field trial lasting up to 3 min, immediately followed by a novel-object trial lasting up to 2 min [19,20,21]. Short transitional periods (e.g., waiting and recovery intervals) were included before and after the two focal phases, resulting in an overall sequence corresponding to the “3 + 2 min” structure depicted in the arena schematic.

### 2.4. Behavioural Assays and Personality Trait Quantification

**Figure 1:**
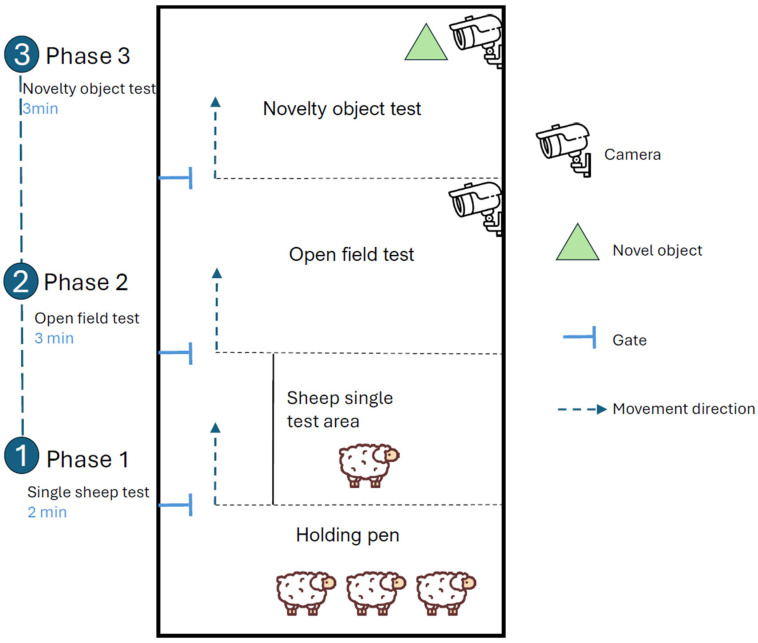
Schematic diagram of the experimental arena and surrounding test arrangement, showing the holding area, pretest compartment, open-field zone, novelty zone, resting zone, and the direction of animal movement during testing.

Behavioural assays were conducted in a fenced experimental arena that was subdivided into five functional zones: a group-holding (waiting) area, an individual pretest compartment, an open-field zone, a novel-object (novelty) zone, and a resting zone. Adjacent zones were connected by gates that allowed for unidirectional movement of the focal animal, preventing backward transitions and ensuring that the sequence, duration, and order of exposure were standardised across individuals. The spatial arrangement of these zones is shown in Figure 1.

Sheep were tested individually, with only one focal animal present in the arena at any time. Non-focal conspecifics remained in the holding area outside the test zones, minimising direct social interactions while maintaining a consistent husbandry background. Because sheep are highly social animals, this individual testing procedure may evoke behavioural responses related not only to exploration or novelty, but also to temporary social isolation; therefore, the interpretation of each behavioural variable was made cautiously and on the basis of its operational definition [20,22]. The dimensions and arrangement of the testing zones were selected to provide the focal animals with sufficient space for normal locomotor movement during each phase of the test while maintaining a standardised testing context [21].

Behaviour was recorded using elevated video cameras (Sony, PCM-D100, Tokyo, Japan) positioned to provide complete coverage of all relevant zones, following the general behavioural recording framework used in our previous Tibetan sheep study [21]. Video recordings were obtained in a colour format and stored using a portable hard drive (Lenovo, Shenzhen, China). The video recordings were subsequently analysed to extract movement-based metrics (e.g., total distance travelled) and event-based measures (e.g., latencies to enter specific zones or to contact novel objects). When automated tracking was not feasible under field conditions, behaviours were scored manually from the video, using predefined ethograms and time-stamped event logging. The scored observations were then transferred to structured data sheets for subsequent statistical analysis. The ethogram defined the specific behavioural events and latency-based variables recorded during each phase of the test. All video scoring was conducted blind to the physiological data of focal individuals to reduce observer expectation bias. No single composite variable termed “behavioural responsiveness” was used in the present study. Instead, the behavioural responses were quantified using separate recorded variables, including latency-based responses, locomotor activity, vocalisation, novelty response, and docility-related behaviour.

#### 2.4.1. Boldness/Shyness

Boldness was defined as an individual’s propensity to enter and remain in an exposed environment, and was quantified using the latency to enter the open-field zone. To avoid conceptual overlap, post-entry movement within the arena was not interpreted as boldness itself, but as a separate measure of locomotor activity/exploration. Shorter latencies were interpreted as greater boldness, whereas longer latencies indicated greater caution or shyness. At the beginning of each trial, a focal sheep was guided from the pretest compartment to the entrance gate of the open-field zone. Following the gate opening, the time until the animal fully crossed into the open-field zone was recorded. This latency-based measure was used as the operational index of boldness in this study. Shorter latencies were interpreted as greater boldness, while longer latencies indicated increased caution (shyness) [2,19].

To minimise the potential confounding effects of transient disturbances (e.g., sudden noise, strong wind, or agitation among nearby animals), all transitions were conducted slowly and consistently. The trials were temporarily paused and, if necessary, repeated if acute disturbances occurred during the entry phase. If an individual failed to enter the open-field zone within the predefined maximum duration, the latency was right-censored at the phase limit, and the animal was gently guided forward to maintain consistency in the testing sequence.

#### 2.4.2. Exploration

Exploration was quantified as locomotor activity within the open-field zone, a demarcated unfamiliar area that was designed to elicit spontaneous movement and scanning behaviour [19,20,21]. Accordingly, maternal activity in this study was measured as locomotor movement expressed during this open-field phase. Following entry into the open-field zone, the focal animal’s movement trajectory was recorded for up to 3 min. The exploration metrics included the total distance travelled and, where video quality permitted, movement-structure variables (e.g., number and duration of movement bouts). In addition, the number of vocalisations emitted during the open-field phase was recorded as a separate behavioural variable, rather than being incorporated into a single broad measure of “behavioural responsiveness”. In this study, exploration was therefore defined primarily from locomotor behaviour within the arena, rather than from vocal output. A greater total distance travelled was interpreted as a higher exploratory tendency; however, this measure was interpreted cautiously because locomotor responses in individually tested sheep may reflect both exploratory movement and agitation-related activity under temporary social isolation. Therefore, the total distance travelled was not treated as a direct measure of boldness in the present study [19,20]. However, the total distance travelled was interpreted cautiously, because locomotor responses in individually tested sheep may reflect both exploratory movement and agitation-related activity under temporary social isolation. The present study quantified the overall locomotor movement within the open-field zone, but did not further subdivide this measure into calm walking versus agitation-driven running. Accordingly, post-entry movement was interpreted conservatively as locomotor activity/exploration in the test context and was not treated as a direct measure of boldness.

Vocalisation was recorded separately and was not treated as a direct measure of exploration. Instead, vocal responses were interpreted more cautiously as an auxiliary indicator of emotional reactivity under temporary individual isolation, because sheep may vocalise when separated from conspecifics or exposed to potentially stressful test conditions [20,22]. This interpretation is also consistent with our previous work in Tibetan sheep, in which vocalisation was analysed as a distinct behavioural variable associated with arousal/stress-related responsiveness rather than exploratory movement [21]. The present study quantified the overall locomotor movement within the open-field zone, but did not further subdivide this measure into calm walking versus agitation-driven running. Accordingly, post-entry movement was interpreted conservatively as locomotor activity/exploration under the test context.

#### 2.4.3. Novelty Response

Novelty response was assessed in a dedicated novelty zone containing an unfamiliar stationary object used as the novel stimulus [19,21]. The novel objects included an orange-and-white parking cone, a wet-floor sign, and a yellow man-shaped “danger” sign, which were used at different testing stages. The novelty-related behaviour was quantified by (i) latency to first contact with any novel object and (ii) behavioural engagement in proximity to the objects [21]. Latency was defined as the time from entry into the novelty zone until the first physical contact (e.g., sniffing, nudging, or touching) with the stationary novel object. If no contact occurred within the maximum novelty-phase duration (2 min), latency was censored at 120 s. All objects were placed before the focal animal entered the novelty zone and remained stationary throughout the trial. Therefore, this assay was designed to measure the response to an unfamiliar stationary object, rather than a startled reaction to sudden movement.

The novel object remained stationary throughout the trial, such that the assay measured the animal’s response to an unfamiliar stationary object rather than to sudden object movement [19]. To maintain object novelty across repeated measurements and minimise habituation, objects were cleaned between trials and stored out of sight of the holding pens. The object positions were fixed within each testing day to ensure comparable spatial exposure among individuals but were rotated across test days when multiple sessions were conducted to balance potential side biases.

#### 2.4.4. Docility Test

Docility was defined as an individual’s tendency to remain calm and settle following exposure to novelty. After the novelty phase, the gate to the resting zone was opened, and the latency to voluntarily enter the resting zone was recorded. This variable was operationally interpreted from the animal’s latency to enter and settle under the standardised post-test condition. Individuals that entered promptly and settled quickly were considered to be more behaviourally calm in this context. Because no separate ethogram was used to score the agitation intensity, this measure was not interpreted as a formal agitation score, but rather as a settling- or recovery-related behavioural response. Where the video resolution allowed it, additional settling indices (e.g., stationary time within the resting zone) were also recorded. Because behaviour in the resting area may reflect both post-test recovery and the animal’s perception of that compartment as relatively sheltered or less aversive, this variable was interpreted cautiously and not as a comprehensive measure of docility alone [19,20]. For statistical analyses, behavioural variables were retained in their original measured forms (e.g., OF.call, NO.call, NO.explo), which were subsequently interpreted within broader personality dimensions.

### 2.5. Physiological Measurements

Physiological data were collected on the same day as the behavioural testing to maintain temporal alignment at the individual level. Measurements were scheduled in the afternoon, following the completion of the behavioural assay sequence, and focused on body condition, autonomic reactivity, and endocrine and microbial variables that were potentially associated with personality and growth.

#### 2.5.1. Body Condition and Morphometrics

Body mass and standard morphometric traits were recorded for all individuals, including body length, withers height, and chest circumference. Body mass was measured using a calibrated livestock scale. Linear dimensions were recorded with a measuring tape and measuring stick, with animals positioned squarely on level ground to ensure consistent posture during measurement.

#### 2.5.2. Struggle Rate, Respiration Rate, and Heart Rate

To characterise stress reactivity and autonomic function, the struggle rate, respiration rate, and heart rate were measured in a brief, standardised handling procedure under light manual restraint. During this phase, each animal was gently restrained by trained personnel for short-term physiological measurement and sample collection, using the same handling sequence for all individuals. Struggle-related behaviour was quantified during the handling phase, based on the animal’s escape-related movements expressed under light manual restraint within a fixed observation interval. The respiration rate was measured by counting thoracic excursions over a defined period, and the heart rate was assessed immediately after restraint using field-appropriate methods. No separate formal ordinal scoring system was applied beyond the original handling-based recording procedure; accordingly, this variable was interpreted as a direct handling-response measure, rather than as a composite temperament score. When recording equipment was available, cardiac beats were obtained from short-duration thoracic recordings and subsequently quantified from the waveform using standard acoustic and physiological analysis procedures. All handling was performed by experienced personnel using minimal restraint to minimise unnecessary disturbances. Accordingly, the struggle-related behaviour in the present study should be understood specifically as a response expressed during the standardised handling phase.

#### 2.5.3. Hormones

Fresh faecal samples were collected immediately after defecation into sterile containers. Samples were kept chilled in the field and were transferred to frozen storage (−20 °C) as soon as practicable for subsequent quantification of glucocorticoid-related hormone measures (e.g., faecal glucocorticoid metabolites) using validated immunoassay protocols.

### 2.6. Statistical Analyses

#### 2.6.1. Repeatability of Behavioural and Physiological Traits

Repeatability (intra-class correlation; R) was used to quantify the consistency of behavioural and physiological traits across repeated measurements. Repeatability was estimated in rptR as the intra-class correlation derived from Gaussian mixed-effects models, with individual identity (ID) fitted as a random intercept (1|ID). Sex was included as a fixed factor, and the measurement date was included as a fixed covariate to account for temporal trends (the date was converted to a numeric scale, e.g., days since the first measurement). Only individuals with repeated observations (*n* > 1) were retained for repeatability estimation.

Uncertainty was quantified using parametric bootstrapping (nboot = 1000) to derive 95% confidence intervals for R. Statistical support for repeatability was assessed using likelihood ratio tests (LRT), comparing models with and without the ID random effect; these LRT-based *p* values are reported in the main text. In addition, permutation tests (npermut = 1000) were conducted as a robustness check, and permutation-based *p* values are provided in Table S1.

#### 2.6.2. Behavioural Syndromes and Trait Covariation

Correlations among personality and physiological traits were quantified to test for behavioural syndromes. To control for potential confounding by sex and age, each trait was first regressed on sex and age using linear models, and correlations were then calculated for the resulting residuals (i.e., covariate-adjusted residual correlations). Pairwise Pearson correlation coefficients (r) and the associated *p* values were obtained using Hmisc::rcorr. The correlation structure was visualised as a heat map with the effect size (r) displayed in each cell.

#### 2.6.3. Effects of Maternal and Offspring Traits on Offspring Growth

The effects of maternal and offspring traits on offspring growth were analysed using linear mixed-effects models. Offspring growth was summarised as a composite score derived from the first principal component (PC1) of four morphometric traits (weightO, lengthO, heightO, and chestO) after centring and scaling, termed growth_composite_pca_O. PC1 scores were calculated from complete cases of the four growth traits and assigned back to the analysis dataset used for model fitting.

To address maternal versus offspring predictors separately, we fitted two independent candidate model sets and conducted model selection within each set. In the maternal candidate model set, growth_composite_pca_O was modelled using maternal behavioural and physiological predictors as candidate fixed effects. In the offspring candidate model set, the same response variable was modelled using offspring behavioural and physiological predictors as candidate fixed effects. In both model sets, mixed-effects models were fitted using nlme, with random intercepts for ewe identity and offspring identity nested within ewe identity (1|IDF/IDO). Control variables (sexO, ageO, sexF, and ageF) were included as fixed effects in the global model and were forced into all candidate models (i.e., not subject to variable selection), such that model selection targeted only the focal maternal or offspring predictor set.

Prior to model selection, continuous candidate predictors were z-transformed (mean = 0, SD = 1). Predictors with all missing values or zero variance were excluded before selection. Variable selection proceeded in two stages: (1) LASSO was used as a preliminary screening step to reduce model complexity prior to information-theoretic model selection. LASSO regression (glmnet; α = 1) with 10-fold cross-validation was used as a screening step, retaining predictors with non-zero coefficients at λmin; (2) information-theoretic model selection was performed using MuMIn::dredge on the corresponding global mixed-effects model fitted by maximum likelihood (ML), ranking candidate models by AICc while forcing covariates to remain in all models. The best-supported model (lowest AICc) was refitted using the restricted maximum likelihood (REML) for parameter estimation. Variable importance was summarised as the sum of Akaike weights across the dredged model set (sw), and the marginal effects of top-ranked predictors were visualised using ggeffects while holding other covariates constant. All analyses were conducted in R (version 4.0.3).

#### 2.6.4. Random Forest Analysis

To complement the AICc-based mixed-effects model selection, Random Forest (RF) regression was used to evaluate the relative importance of predictors for the offspring growth composite. Offspring growth was summarised as a composite trait using the first principal component (PC1) derived from four morphometric measures (weightO, lengthO, heightO, and chestO) after centring and scaling. PC1 scores were calculated from complete cases of the four growth traits and then assigned back to the full dataset by the row index.

Two separate RF models were fitted. In the maternal RF, PC1 was the response variable, and the candidate predictors comprised maternal behavioural and physiological traits (boldnessF, OF.callF, explorationF, NO.callF, NO.exploF, escapeF, docilityF, NOF, weightF, heightF, chestF, struggleF, breathF, HRF, and CORTF). For the offspring analysis, PC1 was modelled using offspring predictors (boldnessO, OF.callO, explorationO, NO.callO, NO.exploO, escapeO, docilityO, NOO, struggleO, breathO, HRO, and CORTO). In both analyses, control variables (sexO, ageO, sexF, and ageF) were included in model training to adjust for these factors, but variable-importance results were reported only for the focal predictor set.

RF models were implemented in R using the randomForest package with permutation-based importance enabled. Model fitting was performed on complete cases for the response and all predictors included in training. The variable importance was quantified using permutation importance (increase in prediction error when a variable is permuted) and decrease in node purity (total reduction in the residual sum of squares attributed to splits on a variable). For visualisation, the importance values were extracted from the fitted RF objects and plotted for the focal predictors only.

## 3. Results

### 3.1. Repeatability of Behavioural Traits and Identification of Stable Individual Differences

Repeatability analyses revealed that only a subset of behavioural traits exhibited consistent among-individual variation (Table S1). Table S1 is provided in the Supplementary Materials. Vocalisation-related traits showed the highest repeatability (*REP*), with the number of calls in the open-field test (OF.call; *REP* = 0.529, 95% CI = [0.376, 0.672]) and in the novel-object test (NO.call; *REP* = 0.665, 95% CI = [0.492, 0.798]) indicating that these vocalisation measures were among the most consistent behavioural variables.

Exploration-related behaviours showed moderate repeatability. Exploration in the open-field test (*REP* = 0.229, 95% CI = [0.043, 0.380]) and exploration of novel objects (NO.explo; *REP* = 0.454, 95% CI = [0.286, 0.596]) were both significantly repeatable, although at lower levels than vocalisation traits. In contrast, boldness showed only low repeatability (*REP* = 0.164, 95% CI = [0, 0.319]), indicating limited temporal consistency.

Several behavioural measures did not exhibit significant repeatability. Escape (*REP* = 0.131, 95% CI = [0, 0.470]), docility (*REP* = 0.035, 95% CI = [0, 0.170]), and time spent contacting novel objects (NO; *REP* = 0.029, 95% CI = [0, 0.227]) showed very low repeatability, suggesting that these variables may reflect short-term behavioural states rather than stable individual traits.

In contrast to the behavioural measures, the physiological traits showed no evidence of repeatability. Struggle (link-scale *REP* = 0.075, *p* = 0.29; Table S1) and respiration rate (*REP* = 0.064, 95% CI = [0, 0.212]) showed low repeatability, while heart rate (HR; *REP* = 0, 95% CI = [0, 0.152]) and cortisol (CORT; *REP* = 0, 95% CI = [0, 0.316]) showed no repeatability.

### 3.2. Correlations Among Behavioural, Physiological, and Morphometric Traits

**Figure 2:**
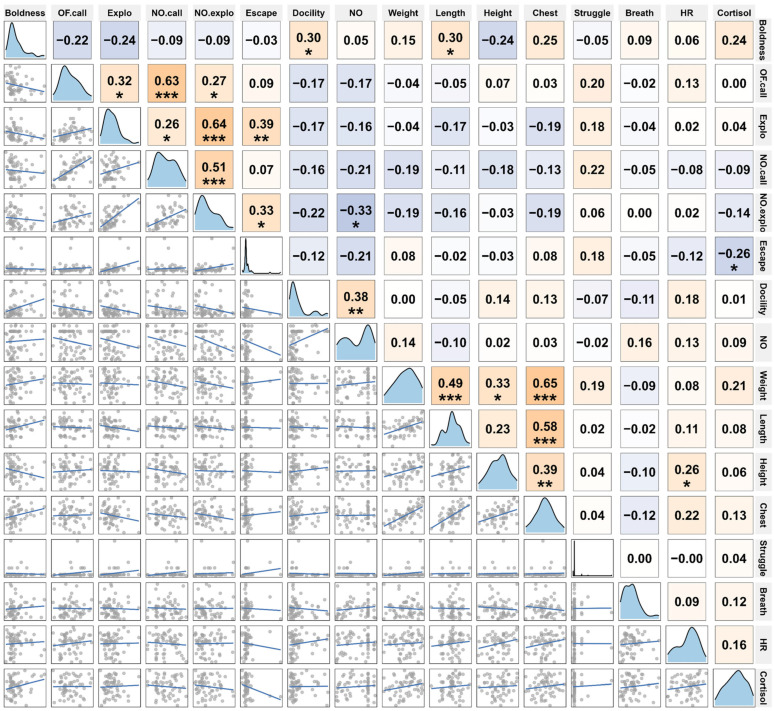
Correlation matrix of behavioural, physiological, and morphometric traits. Pairwise correlations among traits based on sex- and age-adjusted residuals. Colour intensity indicates the strength and direction of correlations, and symbols denote statistical significance (* *p* < 0.05, ** *p* < 0.01, *** *p* < 0.001). HR, heart rate; Explo, exploration.

Correlation analyses based on sex- and age-adjusted residuals revealed several structured relationships among behavioural, physiological, and morphometric traits (Figure 2).

Among behavioural traits, boldness was negatively correlated with exploration (r = −0.33, *p* < 0.05), indicating that individuals entering the open field more rapidly did not necessarily exhibit higher locomotor activity. In contrast, vocalisation traits were positively associated across contexts, with OF.call correlated with NO.call (r = 0.38, *p* < 0.01), suggesting consistent individual differences in vocal responsiveness.

Physiological traits showed strong internal associations. Respiration rate (breath) was positively correlated with heart rate (HR; r = 0.69, *p* < 0.001), indicating coordinated physiological responses. In addition, HR was strongly positively correlated with morphometric traits, including weight, length, height, and chest circumference (r = 0.89, 0.84, 0.77, and 0.73, respectively; all *p* < 0.001), suggesting that larger individuals tended to exhibit higher heart rates.

Morphometric traits were also closely correlated with each other. Chest circumference showed strong positive associations with weight, length, and height (r = 0.79–0.69, all *p* < 0.001), reflecting consistent scaling relationships among body-size variables.

In contrast, cortisol (CORT) showed negligible correlations with other traits, including heart rate (r = 0.04, *p* = 0.75), indicating a limited association between glucocorticoid levels and behavioural or physiological variation under the conditions of this study.

### 3.3. Effects of Maternal Factors on Offspring Growth Traits

Model selection based on the AICc identified Model 127 as the best-supported mixed-effects model for the offspring growth composite (growth_composite_pca_O; PC1 derived from weightO, lengthO, heightO, and chestO), with an AICc value of 439.57, ΔAICc = 0, and an AICc weight of 0.0729 (Table S2). Table S2 is provided in the Supplementary Materials. Across the five top-ranked models (lowest AICc values; Table 1), a consistent set of maternal predictors was retained, including breathF, chestF, docilityF, escapeF, HRF, and NO.callF; boldnessF was not included in the best-supported model.

In the top-ranked model, offspring growth was positively associated with maternal breathF (β = 0.394), escapeF (β = 0.237), and HRF (β = 0.261), while negative associations were observed with maternal chestF (β = −0.585), docilityF (β = −0.244), and NO.callF (β = −0.348) (Table 1). These patterns were consistent across the highest-ranking candidate models, indicating that both behavioural responsiveness-related traits and morphological characteristics contributed to the variation in offspring growth.

**Figure 3:**
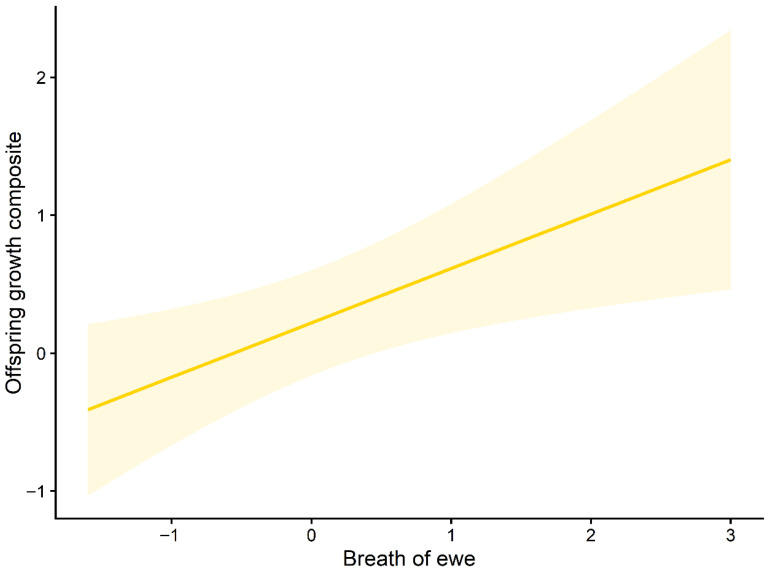
Marginal effect of the maternal respiration rate (breathF) on offspring growth. Predicted relationship between maternal respiration rate and the offspring growth composite (PC1) based on the top-ranked mixed-effects model. The solid line represents fitted values, and the shaded area indicates 95% confidence intervals.

**Figure 4:**
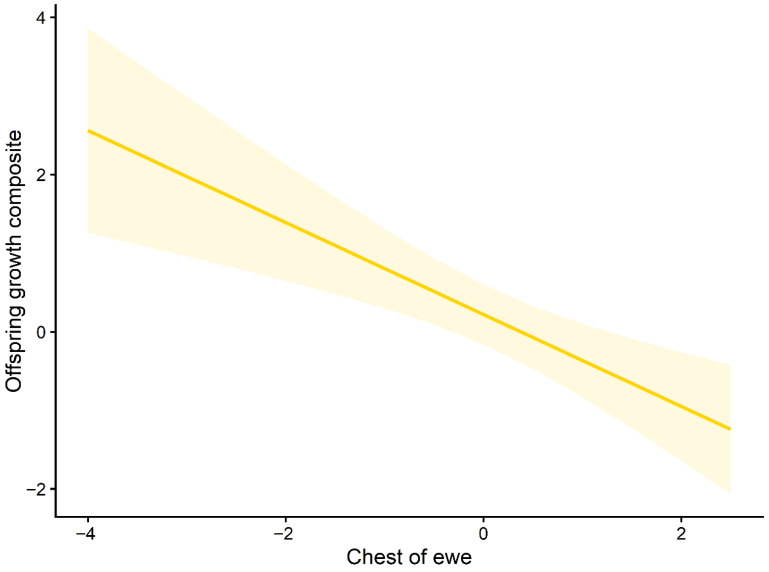
Marginal effect of maternal chest circumference (chestF) on offspring growth. Predicted relationship between maternal chest circumference and the offspring growth composite (PC1) based on the top-ranked mixed-effects model. The solid line represents fitted values, and the shaded area indicates 95% confidence intervals.

**Figure 5:**
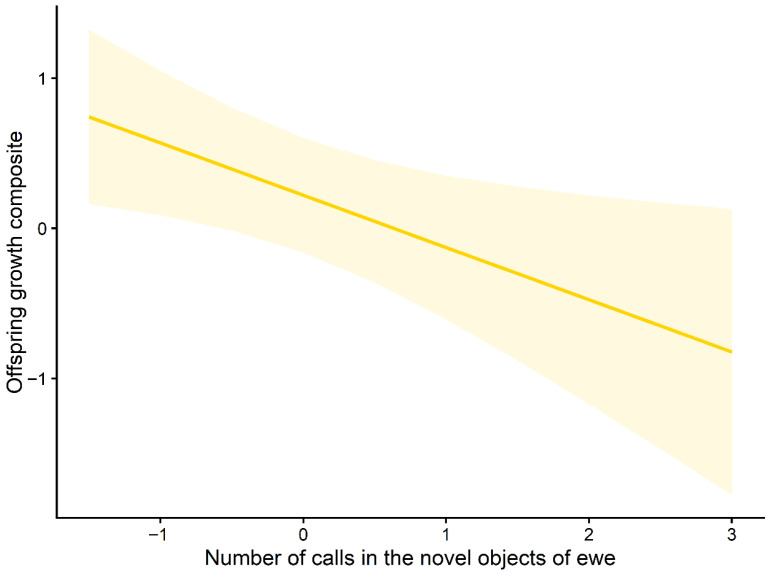
Marginal effect of maternal vocalisation in the novel-object test (NO.callF) on offspring growth. Predicted relationship between the number of calls emitted by ewes in the novel-object test and the offspring growth composite (PC1) based on the top-ranked mixed-effects model. The solid line represents fitted values, and the shaded area indicates 95% confidence intervals.

Marginal effects plots further illustrated these relationships. The predicted offspring growth increased with higher maternal breathF (Figure 3), whereas growth decreased across the observed ranges of maternal chestF and maternal NO.callF (Figure 4 and Figure 5). The full set of candidate models is provided in the Supplementary Materials (Table S2).

### 3.4. Effects of Offspring Behavioural and Physiological Traits on Growth

Model selection based on the AICc identified Model 494 as the best-supported mixed-effects model for the offspring growth composite (growth_composite_pca_O; PC1 derived from weightO, lengthO, heightO, and chestO), with an AICc value of 416.90, ΔAICc = 0, and an AICc weight of 0.0879 (Table S3). Table S3 is provided in the Supplementary Materials. Across the five top-ranked models (lowest AICc values; Table 2), a consistent set of offspring behavioural and physiological predictors was retained, whereas breathO, escapeO, and struggleO were not included in the best-supported model.

Within the top-ranked model, offspring growth showed negative associations with behavioural traits, including boldnessO (β = −0.455), docilityO (β = −0.432), explorationO (β = −0.346), NO.exploO (β = −0.484), and OF.callO (β = −0.360) (Table 2). In contrast, physiological traits showed positive associations with growth, with heart rate (HRO; β = 0.434) and cortisol (CORTO; β = 0.250) both contributing positively.

**Figure 6:**
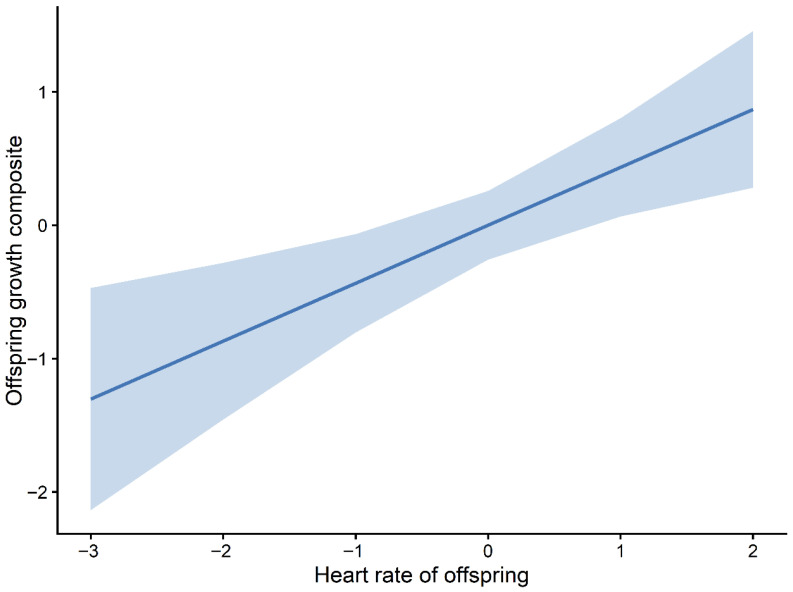
Marginal effect of the offspring heart rate (HRO) on growth. Predicted relationship between offspring heart rate and the offspring growth composite (PC1) based on the top-ranked mixed-effects model. The solid line represents fitted values, and the shaded area indicates 95% confidence intervals.

**Figure 7:**
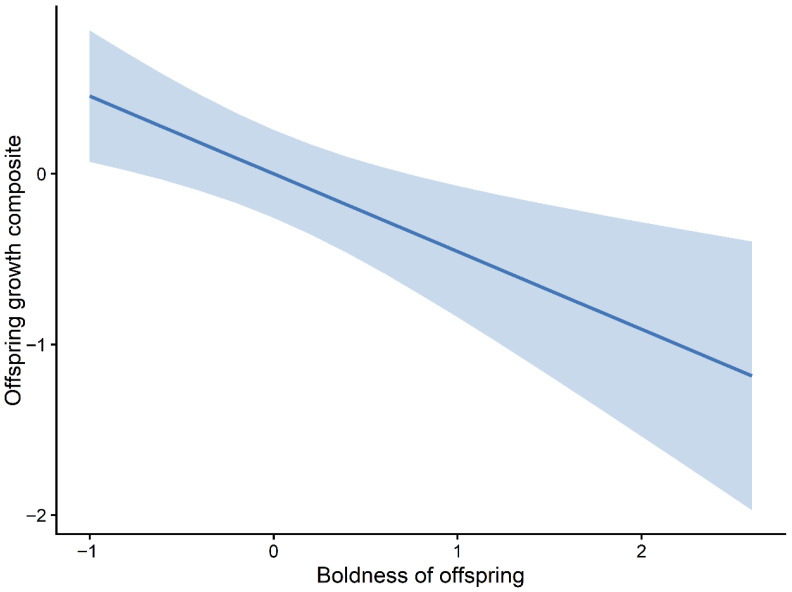
Marginal effect of offspring exploration in the novel-object test (NO.exploO) on growth. Predicted relationship between offspring exploration behaviour in the novel-object test and the offspring growth composite (PC1) based on the top-ranked mixed-effects model. The solid line represents fitted values, and the shaded area indicates 95% confidence intervals.

**Figure 8:**
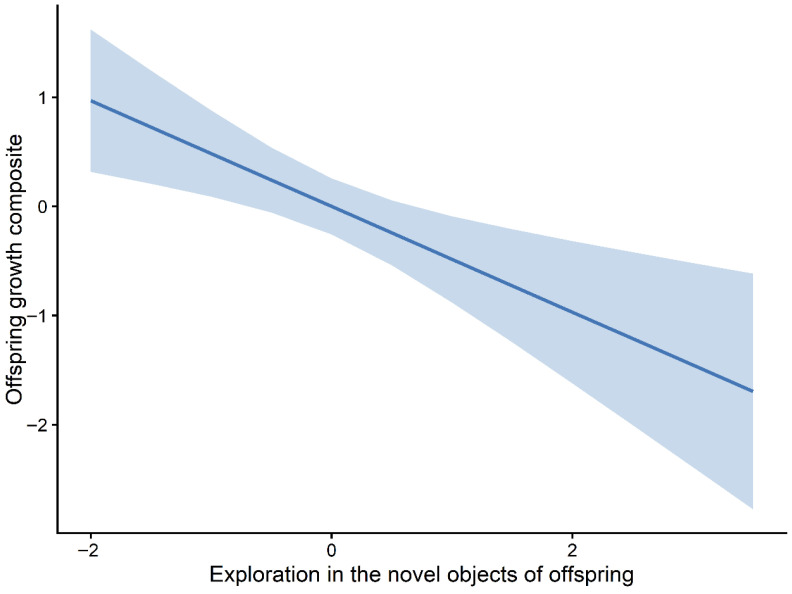
Marginal effect of offspring boldness (boldnessO) on growth. Predicted relationship between offspring boldness and offspring growth composite (PC1) based on the top-ranked mixed-effects model. The solid line represents fitted values, and the shaded area indicates 95% confidence intervals.

These patterns were broadly consistent across the highest-ranking candidate models, suggesting a divergence between behavioural and physiological correlates of growth. Marginal effects plots further illustrated these relationships, with the predicted offspring growth increasing with HRO (Figure 6) and decreasing across the observed ranges of NO.exploO and boldnessO (Figure 7 and Figure 8). The full set of candidate models is provided in the Supplementary Materials (Table S3).

### 3.5. Predictor Importance Based on Random Forest Models

Random Forest (RF) analyses were used to provide an additional assessment of predictor importance for the offspring growth composite, based on the same PC1 response variable. Control variables were included during model fitting but are not presented in the importance summaries (Figures S1 and S2). Details of Figures S1 and S2 can be found in the Supplementary Materials.

In the maternal RF model, permutation-based importance (an increase in prediction error following variable permutation) was the highest for NOF and escapeF, followed by NO.callF and chestF (Figure S1a). The decrease in node purity showed a broadly similar pattern, again identifying NOF and escapeF as the most influential variables in model splits (Figure S1b). Several predictors, including weightF, breathF, heightF, and CORTF, showed near-zero or negative permutation importance, indicating that permuting these variables did not increase the prediction error and, in some cases, slightly reduced it.

In the offspring RF model, the permutation importance was the highest for CORTO, NOO, and escapeO (Figure S2a). The decrease in the node purity similarly highlighted CORTO and NOO as key predictors contributing to reductions in residual variance (Figure S2b). In contrast, explorationO and boldnessO showed negative permutation importance, suggesting that these variables contributed little to predictive performance in the fitted model.

## 4. Discussion

### 4.1. Behavioural Repeatability and the Structure of Personality in Tibetan Sheep

Tibetan sheep showed repeatable individual differences in some behavioural measures, but this pattern was not uniform across traits. In this study, vocalisation was therefore interpreted as a distinct behavioural response reflecting emotional reactivity under temporary isolation, rather than as a direct indicator of exploratory behaviour [20,21,22]. Vocalisation in both the open-field and novel-object tests, together with exploration-related measures, showed moderate to high repeatability, whereas boldness and docility were only weakly repeatable. This distinction matters because repeatability is a basic criterion for separating stable individual differences from short-term behavioural states. In this context, the present results suggest that only a subset of commonly used behavioural measures in sheep may reliably capture such differences [2,7].

The relatively high repeatability of vocalisation is noteworthy. In sheep, vocal behaviour is often associated with social reactivity and separation-related arousal and may therefore provide a consistent indicator of individual responsiveness under controlled testing conditions. While it is not possible to infer underlying mechanisms directly, the consistency of vocal responses across contexts suggests that this trait may reflect a stable axis of behavioural variation within the studied population. In contrast, boldness, as estimated from latency measures, appeared less consistent over time. This may reflect greater sensitivity to transient factors such as environmental disturbances or handling context, which could obscure underlying individual differences, even if they are present [2,7].

The absence of repeatability in docility warrants particular caution. Although docility is often treated as a core component of temperament in livestock studies, the present results suggest that, in this system, it may instead reflect short-term behavioural adjustments following novelty exposure or handling. In other words, responses classified as “docile” in one trial may not arise from the same underlying processes in another. This highlights a broader issue in studies of domestic ungulates: not all behaviourally plausible measures necessarily meet the criteria required to be considered personality-related traits, and careful trait selection remains essential. These findings indicate that stable behavioural variation in Tibetan sheep is detectable but unevenly distributed across traits. Rather than representing a uniform personality structure, behavioural variation appears to be composed of traits that differ in their degree of temporal stability. This pattern is consistent with a developmental perspective on animal behaviour in which some traits are more canalised, while others remain sensitive to context or age-related changes [7,8].

### 4.2. Trait Covariation and the Weak Integration of Behavioural Syndromes

The correlations among the behavioural traits were generally weak, and the observed relationships changed with context, rather than forming a tightly integrated behavioural syndrome. Notably, boldness was negatively correlated with exploration. Although this contrasts with the positive association that is commonly reported between these traits, it is not necessarily inconsistent with ecological expectations, as correlations among behavioural traits can vary depending on environmental conditions and ecological context [1,23,24]. In the present system, latency to enter an exposed area and subsequent movement within that area may reflect distinct behavioural processes. The former likely captures hesitation at the boundary between safety and exposure, whereas the latter may reflect locomotor activity or behavioural arousal once inside the arena. Under semi-captive conditions, where predation risk is minimal and resource access is externally regulated, these components may not covary in a consistent direction.

In contrast, vocalisation showed more consistent covariation across contexts. The positive association between open-field and novel-object vocalisation suggests that individual differences in vocal responsiveness are expressed across test situations. This pattern is consistent with previous work in sheep showing that vocalisation can exhibit relatively high repeatability and may reflect stable behavioural tendencies such as sociability or reactivity to isolation [25,26]. Short-term seclusion has been shown to alter behavioural welfare indicators in sheep, with increased activity and vocalisation interpreted as responses to emotional stress and social separation [27]. As vocal behaviour in sheep is closely linked to social dependence and arousal, individuals that vocalise more readily in one mildly stressful context may show similar responses in others. In this sense, vocalisation may represent a relatively stable behavioural axis in Tibetan sheep compared to traits such as boldness or docility.

The physiological correlation structure provided further context for interpreting these patterns. Heart rate and respiration rate covaried strongly, as expected, and heart rate was closely associated with morphometric traits. These relationships likely reflect shared scaling processes, rather than coordinated behavioural variation, as physiological and morphological traits are often linked through body size-related constraints. In contrast, cortisol showed little association with either behavioural or physiological measures. This weak coupling suggests that the endocrine variation, as captured by faecal glucocorticoids, does not align closely with the behavioural variation measured in arena tests, a pattern that was also reported in other studies where behavioural and endocrine responses are only weakly integrated [2,28,29]. Our findings indicate that behavioural and physiological traits in Tibetan sheep are only weakly integrated. Rather than forming a cohesive behavioural syndrome, trait variation appears to be structured along partially independent axes. This is consistent with broader theoretical work showing that behavioural syndromes are not universal, but can vary in strength and structure depending on their ecological conditions, developmental processes, and measurement context [1,23,24].

### 4.3. Maternal Traits and Offspring Growth

The most notable result of this study is that maternal behavioural and physiological traits were associated with offspring growth, although these relationships were neither uniform nor easily interpreted in a single direction. In the best-supported models, offspring growth was positively associated with the maternal respiration rate, escape behaviour, and heart rate but negatively associated with the maternal chest circumference, docility, and novel-object calling. This pattern is broadly compatible with the parental effects theory, in which the maternal phenotype can shape offspring phenotype through both genetic and non-genetic pathways [6,9]. In our system, however, the direction of these associations varied among traits, suggesting that ecological and management conditions shape how maternal characteristics translate into offspring growth [13].

The negative association between maternal docility and offspring growth warrants careful interpretation. In livestock systems, docility is often assumed to be beneficial; however, this assumption may not hold across all environmental contexts. Under semi-captive plateau conditions, more docile individuals may not necessarily achieve higher effective resource acquisition or allocation to offspring. One possibility is that lower behavioural responsiveness could reduce flexibility in foraging or social competition, although this remains speculative. Alternatively, as suggested by the repeatability analysis, docility in this study may reflect short-term behavioural adjustment following testing, rather than a stable maternal trait. Importantly, given its near-zero repeatability, docility in this study should be interpreted cautiously and is more likely to reflect short-term behavioural states rather than a stable personality trait. In sheep, vocalisation during individual testing and struggle-related behaviour during handling may represent more immediate forms of behavioural reactivity linked to temporary isolation and restraint, respectively. These responses may therefore be informative when interpreting maternal responsiveness in this species. In addition, future studies could incorporate a ewe’s willingness to approach a familiar caretaker as a complementary measure of docility, because responses to familiar people may capture a different behavioural dimension from arena-based or handling-based tests alone.

In contrast, the positive associations between offspring growth and maternal escape behaviour, respiration rate, and heart rate suggest that a certain level of behavioural and physiological responsiveness may be linked to improved developmental outcomes. Importantly, these traits should not be interpreted narrowly as indicators of stress. In managed herbivores, short-term increases in respiration or heart rate during behavioural tests may reflect general responsiveness, metabolic activity, or vigilance, rather than chronic stress exposure [28]. If so, more responsive individuals may be better able to adjust their behaviour to variable environmental or social conditions, which could in turn influence resource allocation to offspring. This interpretation is in line with studies linking behavioural variation to energy allocation and life-history trade-offs, although the direction of such relationships differs among ecological contexts [30,31].

These findings also align with previous work on sheep demonstrating that maternal phenotype and behaviour play a central role in shaping early offspring development. Maternal behaviour around parturition, including responsiveness to lambs and mother–offspring interactions, has been shown to influence lamb survival and early growth [14,16,17]. This study extends this perspective by suggesting that maternal effects may not be limited to classical maternal-care behaviours, but also involve broader behavioural and physiological traits that influence offspring development under specific environmental conditions.

### 4.4. Offspring Personality and Growth

Offspring behavioural and physiological traits showed clear associations with growth. In the best-supported models, boldness, docility, exploration, novel-object exploration, and open-field calling were negatively associated with the growth composite, whereas heart rate and cortisol were positively associated. Considered together, these associations suggest a trade-off between behavioural activity and growth, but the magnitude and direction of that trade-off likely depend on the ecological context [31,32].

The negative associations between exploratory and boldness-related behaviours and growth are biologically plausible. Individuals that invest more in locomotion, exploration, or behavioural responsiveness are likely to incur higher energetic costs, which may limit the energy available for somatic growth. Similar trade-offs have been proposed in studies of animal personality, where behavioural variation is linked to differences in energy expenditure, resource acquisition, and life-history strategies [32,33]. In early life stages, when energy intake is constrained by maternal provisioning and limited foraging capacity, such trade-offs may be particularly pronounced.

The positive associations between heart rate and growth may reflect differences in metabolic throughput, rather than stress per se. Higher heart rates are often associated with increased metabolic activity and energy turnover, which can support growth under favourable nutritional conditions [34]. The positive association with cortisol is less intuitive but not necessarily contradictory. Glucocorticoids play a role not only in stress responses but also in energy mobilisation and developmental processes [35]. Moderate elevations in glucocorticoids can facilitate energy availability, particularly in growing individuals, although the direction and magnitude of these effects depend on the context and measurement scale. In the present study, cortisol was assessed from faecal samples, which reflect integrated hormone levels over time rather than acute responses, and endocrine–behaviour relationships are known to be variable and sometimes weak during development [8,36]. These factors warrant caution in interpretation.

More broadly, these results indicate that offspring growth was not determined solely by maternal phenotype but was also associated with the offspring’s own behavioural and physiological characteristics. This suggests that offspring are not passive recipients of maternal effects but active participants in their own developmental trajectories. Interactions between maternal influences and offspring traits may therefore shape growth outcomes in ways that are not fully predictable from maternal characteristics alone, a pattern that has been increasingly recognised in studies of behavioural development and parental effects [5,6].

### 4.5. A Livestock-Specific Perspective on Parental Personality Effects

An important implication of this study is that parental personality effects in livestock may not directly mirror those described in wild systems. In a range of non-domestic taxa, including fishes, parental behavioural traits have been linked to offspring behaviour and performance through both genetic and non-genetic pathways. For example, paternal personality and social status influence offspring activity and development in zebrafish, while parental behavioural traits have been associated with growth and stress-related traits in olive flounder [37,38]. Cross-generational effects of personality have been reported in several taxa, but both their strength and direction vary among ecological settings [6,13].

Our results point to a similar phenomenon in Tibetan sheep, while also suggesting that its consequences differ under semi-captive livestock conditions. In wild systems, behavioural traits such as boldness or activity can enhance resource acquisition, albeit often at the cost of increased predation risk [5,31]. In contrast, under livestock management, the predation risk is largely reduced, movement is constrained, and food availability is more predictable. Under these conditions, the balance of costs and benefits associated with behavioural traits may shift, potentially altering their relationships with offspring development. As a result, behavioural profiles that are advantageous in wild herbivores may not produce equivalent outcomes in managed populations.

This context dependence also provides a framework for interpreting the absence of simple relationships between personality and performance in the present study. Rather than conforming to general rules (e.g., “bolder mothers produce faster-growing offspring”), the observed patterns suggest that the effects of behavioural traits are conditional. In domestic systems, these relationships are likely shaped by interactions among inherited tendencies, maternal effects, environmental constraints, and energy allocation processes. Comparable patterns have been described in other systems, where ecological conditions alter both how behavioural variation is expressed and how strongly it affects performance [23,24].

## 5. Conclusions

Maternal behavioural variation in Tibetan sheep was associated with early offspring growth under semi-captive conditions. These associations were not uniform across traits, and some behavioural measures did not satisfy the criteria required for personality traits. Both maternal and offspring variables were related to growth, indicating that developmental outcomes reflect contributions from maternal effects as well as offspring-specific behavioural and physiological characteristics. More generally, the results suggest that behavioural effects on performance in livestock cannot be interpreted without reference to production context. From a practical perspective, these findings may provide a preliminary basis for future management, selection, and breeding considerations in Tibetan sheep under semi-captive production conditions, although further validation is required before direct on-farm application. Meantime, the present findings should be interpreted with caution, because some behavioural measures did not fully satisfy the criteria required for personality traits, and some responses may be context-dependent. In addition, broader validation under other sheep production systems is still needed. Further work should examine the mechanisms linking maternal responsiveness to offspring development and determine whether similar patterns occur in other sheep production systems.

## Figures and Tables

**Table 1:** Top-ranked mixed-effects models (AICc) for maternal trait predictors of offspring growth composite (PC1).

Model	AICc	Delta	Weight	df	BreathF	ChestF	DocilityF	EscapeF	HRF	NO.callF	SexO	LogLik
127	439.571	0.000	0.073	11	0.394	−0.585	−0.244	0.237	0.261	−0.348	+	−207.492
111	439.688	0.116	0.069	10	0.372	−0.552	−0.279		0.228	−0.356	+	−208.776
79	439.713	0.142	0.068	9	0.357	−0.579	−0.289			−0.363	+	−209.991
119	439.803	0.232	0.065	10	0.412	−0.636		0.272	0.276	−0.306	+	−208.833
95	440.304	0.733	0.051	10	0.374	−0.611	−0.262	0.199		−0.357	+	−209.084

Note: Full candidate model sets (top 50 models) are provided in the Supplementary Materials. Abbreviations: NO.call, number of calls in the novel-object test; HR, heart rate. Suffixes denote individual class: F, ewe; O, offspring. The “+” symbol indicates that the variable was included in the model.

**Table 2:** Top-ranked mixed-effects models (AICc) for offspring trait predictors of offspring growth composite (PC1).

**Model**	**AICc**	**Delta**	**Weight**	**df**	**BoldnessO**	**BreathO**	**CORTO**	**DocilityO**	**EscapeO**	**ExplorationO**	**HRO**	**NO.exploO**	**OF.callO**	**LogLik**
494	416.905	0.000	0.088	11	−0.455		0.249	−0.432		−0.346	0.434	−0.484	−0.360	−196.158
496	418.123	1.218	0.048	12	−0.460	0.147	0. 349	−0.405		−0.335	0.424	−0.482	−0.357	−195.517
490	418.189	1.285	0.046	10	−0.299			−0.429		−0.342	0.428	−0.488	−0.369	−198.027
1006	418.589	1.684	0.038	12	−0.455		0.255	−0.413		−0.331	0.427	−0.6658	−0.371	−195.750
510	418.728	1.823	0.035	12	−0.441		0.241	−0.445	−0.116	−0.331	0.449	−0.473	−0.335	−195.819

Note: Full candidate model sets (top 50 models) are provided in the Supplementary Materials. Abbreviations: OF.call, number of calls in the open-field test; NO.expl, exploration in the novel-object test; HR, heart rate; and CORT, cortisol. Suffixes denote individual class: F, ewe and O, offspring.

## Data Availability

The datasets generated and analysed in this study are available upon request from the corresponding author.

## Supplementary Materials

The following supporting information can be downloaded at: https://www.mdpi.com/article/10.3390/ani16091386/s1, Figure S1: Random Forest variable importance for maternal predictors of offspring growth. Variable importance for maternal predictors of the offspring growth composite (PC1) based on Random Forest models. The models were trained adjusting for control variables (sexO, ageO, sexF, ageF). (a) Permutation importance (increase in prediction error). (b) Decrease in node purity. Abbreviations: OF.call, number of calls in the open-field test; NO.call, number of calls in the novel-object test; NO.explo, exploration in the novel-object test; NO, time spent contacting novel objects; HR, heart rate; and CORT, cortisol. Suffixes denote individual class: F, ewe; O, offspring. Figure S2: Random Forest variable importance for offspring predictors of growth. Variable importance for offspring predictors of the offspring growth composite (PC1) based on Random Forest models. The models were trained adjusting for control variables (sexO, ageO, sexF, ageF). (a) Permutation importance (increase in prediction error). (b) Decrease in node purity. Abbreviations: OF.call, number of calls in the open-field test; NO.call, number of calls in the novel-object test; NO.explo, exploration in the novel-object test; NO, time spent contacting novel objects; HR, heart rate; and CORT, cortisol. Suffixes denote individual class: F, ewe; O, offspring. Table S1: Repeatability (R) of behavioural and physiological traits. Repeatability (intra-class correlation coefficient, R) was estimated using mixed-effects models with individual identity (ID) as a random intercept and sex and measurement date as fixed effects. Values are reported on the link scale where applicable. 95% confidence intervals were obtained by parametric bootstrapping (nboot = 1000). *p*-values in the main text are from likelihood ratio tests (LRT) comparing models with vs. without the ID random effect; permutation-based *p*-values (npermut = 1000) are reported as robustness checks. Abbreviations: OF.call, number of calls in the open-field test; NO.call, number of calls in the novel-object test; NO.explo, exploration in the novel-object test; NO, time spent contacting novel objects; HR, heart rate; CORT, cortisol. Suffixes denote individual class: F, ewe; O, offspring. Table S2: Candidate model set (AICc) for maternal predictors of offspring growth composite (PC1). Abbreviations: OF.call, number of calls in the open-field test; NO.call, number of calls in the novel-object test; NO.explo, exploration in the novel-object test; NO, time spent contacting novel objects; HR, heart rate; CORT, cortisol. Suffixes denote individual class: F, ewe; O, offspring. Table S3: Candidate model set (AICc) for offspring predictors of offspring growth composite (PC1). Abbreviations: OF.call, number of calls in the open-field test; NO.call, number of calls in the novel-object test; NO.explo, exploration in the novel-object test; NO, time spent contacting novel objects; HR, heart rate; CORT, cortisol. Suffixes denote individual class: F, ewe; O, offspring.

## Abbreviations

OF.call	number of calls in the open-field test
NO.call	number of calls in the novel-object test
NO.explo	exploration in the novel-object test
NO	time spent contacting novel objects
HR	heart rate
CORT	cortisol
Suffixes: F	ewe
Suffixes: O	offspring

## References

[1] Sih A., Bell A., Johnson J.C. (2004). Behavioral syndromes: An ecological and evolutionary overview. Trends Ecol. Evol..

[2] Réale D., Reader S.M., Sol D., McDougall P.T., Dingemanse N.J. (2007). Integrating animal temperament within ecology and evolution. Biol. Rev..

[3] Bell A.M., Hankison S.J., Laskowski K.L. (2009). The repeatability of behaviour: A meta-analysis. Anim. Behav..

[4] Smith B.R., Blumstein D.T. (2008). Fitness consequences of personality: A meta-analysis. Behav. Ecol..

[5] Wolf M., Weissing F.J. (2012). Animal personalities: Consequences for ecology and evolution. Trends Ecol. Evol..

[6] Groothuis T., Maestripieri D., Carere C., Maestripieri D. (2013). Parental influences on offspring personality. Animal Personalities: Behavior, Physiology, and Evolution.

[7] Stamps J., Groothuis T.G. (2010). The development of animal personality: Relevance, concepts and perspectives. Biol. Rev. Camb. Philos. Soc..

[8] Günther A., Finkemeier M.-A., Trillmich F. (2014). The ontogeny of personality in the wild guinea pig. Anim. Behav..

[9] Mousseau T.A., Fox C.W. (1998). The adaptive significance of maternal effects. Trends Ecol. Evol..

[10] Marshall D.J., Uller T. (2007). When is a maternal effect adaptive?. Oikos.

[11] Champagne F.A. (2008). Epigenetic mechanisms and the transgenerational effects of maternal care. Front. Neuroendocrinol..

[12] Badyaev A.V., Uller T. (2009). Parental effects in ecology and evolution: Mechanisms, processes and implications. Philos. Trans. R. Soc. Lond. B. Biol. Sci..

[13] Reddon A.R. (2012). Parental effects on animal personality. Behav. Ecol..

[14] Nowak R., Porter R.H., Lévy F., Orgeur P., Schaal B. (2000). Role of mother-young interactions in the survival of offspring in domestic mammals. Rev. Reprod..

[15] Dwyer C. (2008). The welfare of the neonatal lamb. Small Rumin. Res..

[16] Dwyer C.M., Lawrence A.B., Bishop S.C., Lewis M. (2003). Ewe–lamb bonding behaviours at birth are affected by maternal undernutrition in pregnancy. Br. J. Nutr..

[17] Everett-Hincks J., Dodds K. (2008). Management of maternal-offspring behavior to improve lamb survival in easy care sheep systems. J. Anim. Sci..

[18] Beausoleil N.J., Blache D., Stafford K.J., Mellor D.J., Noble A.D. (2008). Exploring the basis of divergent selection for ‘temperament’ in domestic sheep. Appl. Anim. Behav. Sci..

[19] Forkman B., Boissy A., Meunier-Salaün M.-C., Canali E., Jones R. (2007). A critical review of fear tests used on cattle, pigs, sheep, poultry and horses. Physiol. Behav..

[20] Dodd C.L., Pitchford W.S., Edwards J.E.H., Hazel S.J. (2012). Measures of behavioural reactivity and their relationships with production traits in sheep: A review. Appl. Anim. Behav. Sci..

[21] Yu Y., Wang Y., Zhong L., Zhu H., Qu J. (2021). Variations in behavioral and physiological traits in yearling Tibetan sheep (*Ovis aries*). Animals.

[22] Cockram M. (2004). A review of behavioural and physiological responses of sheep to stressors to identify potential behavioural signs of distress. Anim. Welf..

[23] Dingemanse N.J., Wolf M. (2010). Recent models for adaptive personality differences: A review. Philos. Trans. R. Soc. B Biol. Sci..

[24] Sih A., Mathot K.J., Moirón M., Montiglio P.-O., Wolf M., Dingemanse N.J. (2015). Animal personality and state–behaviour feedbacks: A review and guide for empiricists. Trends Ecol. Evol..

[25] Wolf B., McBride S., Lewis R., Davies M., Haresign W. (2008). Estimates of the genetic parameters and repeatability of behavioural traits of sheep in an arena test. Appl. Anim. Behav. Sci..

[26] Atkinson L., Doyle R.E., Woodward A., Jongman E.C. (2022). Exposure to humans after weaning does not reduce the behavioural reactivity of extensively reared Merino lambs. Behav. Process..

[27] De K., Saxena V.K., Balaganur K., Kumar D., Naqvi S.M.K. (2018). Effect of short-term seclusion of sheep on their welfare indicators. J. Vet. Behav..

[28] Koolhaas J., De Boer S., Coppens C., Buwalda B. (2010). Neuroendocrinology of coping styles: Towards understanding the biology of individual variation. Front. Neuroendocrinol..

[29] Carere C., Maestripieri D. (2013). Animal Personalities: Behavior, Physiology, and Evolution.

[30] Biro P.A., Stamps J.A. (2008). Are animal personality traits linked to life-history productivity?. Trends Ecol. Evol..

[31] Réale D., Garant D., Humphries M.M., Bergeron P., Careau V., Montiglio P.-O. (2010). Personality and the emergence of the pace-of-life syndrome concept at the population level. Philos. Trans. R. Soc. B Biol. Sci..

[32] Careau V., Thomas D., Humphries M., Réale D. (2008). Energy metabolism and animal personality. Oikos.

[33] Biro P.A., Stamps J.A. (2010). Do consistent individual differences in metabolic rate promote consistent individual differences in behavior?. Trends Ecol. Evol..

[34] White C.R., Kearney M.R. (2013). Determinants of inter-specific variation in basal metabolic rate. J. Comp. Physiol. B.

[35] Sapolsky R.M., Romero L.M., Munck A.U. (2000). How do glucocorticoids influence stress responses? Integrating permissive, suppressive, stimulatory, and preparative actions. Endocr. Rev..

[36] Goymann W. (2012). On the use of non-invasive hormone research in uncontrolled, natural environments: The problem with sex, diet, metabolic rate and the individual. Methods Ecol. Evol..

[37] Zajitschek S., Herbert-Read J.E., Abbasi N.M., Zajitschek F., Immler S. (2017). Paternal personality and social status influence offspring activity in zebrafish. BMC Evol. Biol..

[38] Yang K., Zhang X., Liu Z., Lu W. (2022). Parental personality influence offspring performance traits in olive flounder (*Paralichthys olivaceus*). Aquaculture.

