# Supplementary material for: Context-Dependent Effects of Maternal Behaviour on Lamb Growth in Tibetan Sheep
Source: Animals (Basel). 2026 May 1;16(9):1386. doi: 10.3390/ani16091386 (PMC13162791; doi:10.3390/ani16091386)
Supplement: Supplementary file 1 [file animals-16-01386-s001.zip › animals-4268266- Figure S1 and Figure S2.pdf]

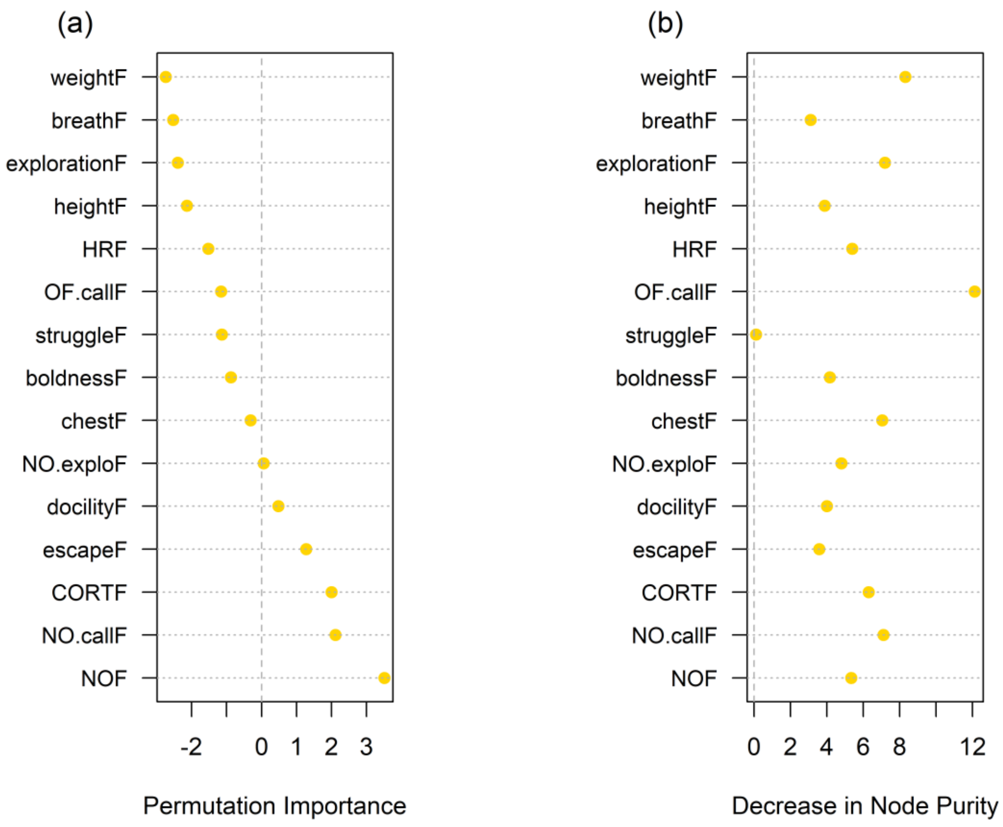

Figure S1. Random Forest variable importance for maternal predictors of offspring growth.Variable importance for maternal predictors of the offspring growth composite (PC1) based on Random Forest models. Control variables (sexO, ageO, sexF, ageF) were included during model training but are not shown. (a) Permutation importance (increase in prediction error). (b) Decrease in node purity. Abbreviations: OF.call, number of calls in the open-field test; NO.call, number of calls in the novel-object test; NO.explo, exploration in the novel-object test; NO, time spent contacting novel objects; HR, heart rate; CORT, cortisol. Suffixes denote individual class: F, ewe; O, offspring.

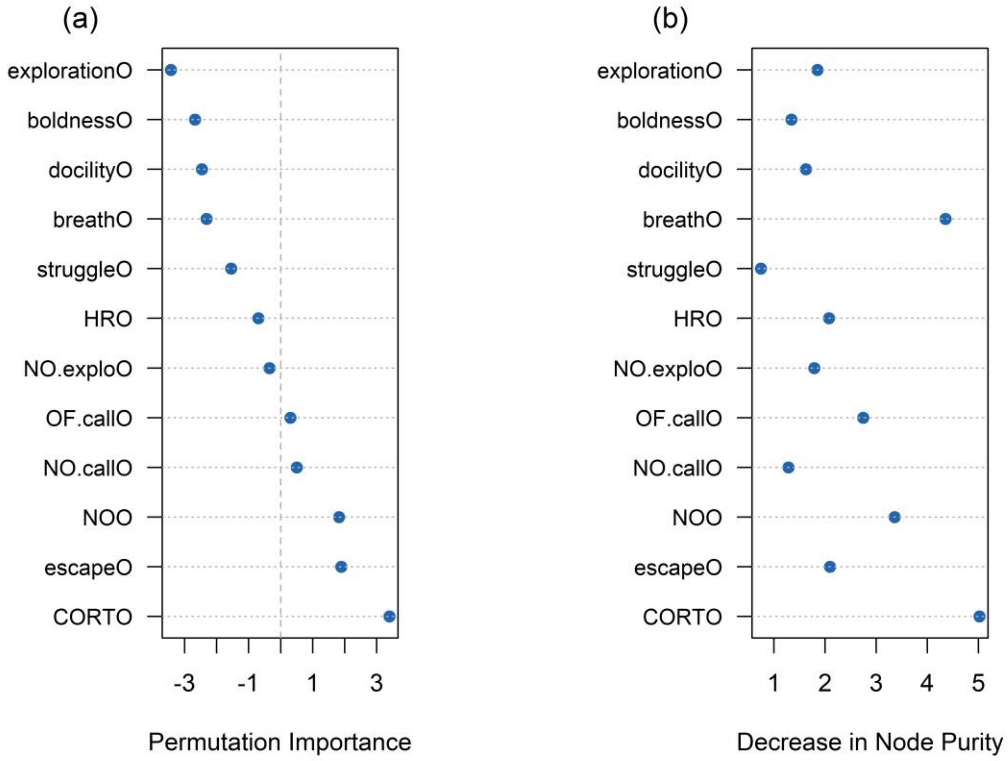

Figure S2. Random Forest variable importance for offspring predictors of growth. Variable importance for offspring predictors of the offspring growth composite (PC1) based on Random Forest models. Control variables (sexO, ageO, sexF, ageF) were included during model training but are not shown. (a) Permutation importance (increase in prediction error). (b) Decrease in node purity. Abbreviations: OF.call, number of calls in the open-field test; NO.call, number of calls in the novel-object test; NO.explo, exploration in the novel-object test; NO, time spent contacting novel objects; HR, heart rate; CORT, cortisol. Suffixes denote individual class: F, ewe; O, offspring.
