# Supplementary material for: Context-Dependent Effects of Maternal Behaviour on Lamb Growth in Tibetan Sheep
Source: Animals (Basel). 2026 May 1;16(9):1386. doi: 10.3390/ani16091386 (PMC13162791; doi:10.3390/ani16091386)
Supplement: Supplementary file 1 [file animals-16-01386-s001.zip › animals-4268266- Table S1.pdf]

Table S1. Repeatability (R) of behavioural and physiological traits.

Repeatability (intra-class correlation coefficient, R) was estimated using mixed-effects models with individual identity (ID) as a random intercept and sex and measurement date as fixed effects. Values are reported on the link scale where applicable. 95% confidence intervals were obtained by parametric bootstrapping (nboot = 1000). P-values in the main text are from likelihood ratio tests (LRT) comparing models with vs. without the ID random effect; permutation-based P-values (npermut = 1000) are reported as robustness checks.

Trait	R	SE	95% CI	P (LRT)	P (Permutation)
boldness	0.164	0.082	0.000–0.319	0.0235	0.231
OF.call	0.529	0.074	0.376–0.672	<0.001	0.001
exploration	0.229	0.084	0.043–0.380	0.00427	0.12
NO.call	0.665	0.082	0.492–0.798	<0.001	0.007
NO.explo	0.454	0.079	0.286–0.596	<0.001	0.016
escape	0.131	0.144	0.000–0.470	0.246	0.375
docility	0.000	0.051	0.000–0.170	0.5	0.568
NO	0.029	0.066	0.000–0.227	0.39	0.489
struggle	0.075	0.019	0.000–0.073	0.29	0.219
breath	0.064	0.063	0.000–0.212	0.214	0.192
HR	0.000	0.044	0.000–0.152	1	0.523
CORT	0.000	0.093	0.000–0.316	1	0.458

Abbreviations: OF.call, number of calls in the open-field test; NO.call, number of calls in the novel-object test; NO.explo, exploration in the novel-object test; NO, time spent contacting novel objects; HR, heart rate; CORT, cortisol. Suffixes denote individual class: F, ewe; O, offspring.
