# Supplementary material for: Context-Dependent Effects of Maternal Behaviour on Lamb Growth in Tibetan Sheep
Source: Animals (Basel). 2026 May 1;16(9):1386. doi: 10.3390/ani16091386 (PMC13162791; doi:10.3390/ani16091386)
Supplement: Supplementary file 1 [file animals-16-01386-s001.zip › animals-4268266- Table S2.pdf]

Table S2. Candidate model set (AICc) for maternal predictors of offspring growth composite (PC1).

Model	AICc	delta	weight	df	(Intercept)	boldnessF	breathF	chestF	docilityF	escapeF	HRF	NO.callF	sexO	logLik
127	439.5713	0.0000000	0.072906326	11	0.2204609		0.3940165	-0.5852079	-0.2443900	0.2365091	0.2609031	-0.3479592	+	-207.4915
111	439.6875	0.1162541	0.068789310	10	0.1952231		0.3718458	-0.5518046	-0.2790542		0.2277808	-0.3562931	+	-208.7758
79	439.7129	0.1416136	0.067922586	9	0.2288091		0.3568231	-0.5791565	-0.2894373			-0.3628979	+	-209.9911
119	439.8029	0.2316463	0.064932761	10	0.2247050		0.4121847	-0.6362573		0.2715812	0.2762769	-0.3061211	+	-208.8335
95	440.3044	0.7331450	0.050531803	10	0.2541751		0.3736537	-0.6106413	-0.2615156	0.1991770		-0.3566888	+	-209.0842
120	440.4858	0.9145067	0.046151157	11	0.2345472	-0.1923196	0.4144230	-0.6140527		0.2544532	0.2799789	-0.3125785	+	-207.9488
112	440.7326	1.1613896	0.040791781	11	0.2057915	-0.1739455	0.3771812	-0.5395011	-0.2489514		0.2345806	-0.3569649	+	-208.0722
103	440.7650	1.1937934	0.040136201	9	0.1958036		0.3892024	-0.6055770			0.2399817	-0.3089382	+	-210.5171
128	440.8683	1.2970180	0.038117230	12	0.2291578	-0.1593141	0.3978412	-0.5722125	-0.2184059	0.2260700	0.2656787	-0.3488585	+	-206.8896
80	440.8844	1.3131173	0.037811631	10	0.2395490	-0.1648486	0.3616779	-0.5689910	-0.2610091			-0.3638483	+	-209.3742
87	440.8962	1.3249599	0.037588398	9	0.2608564		0.3918884	-0.6671327		0.2344996		-0.3122928	+	-210.5827
104	441.0158	1.4445525	0.035406633	10	0.2085157	-0.2145276	0.3934291	-0.5834472			0.2465860	-0.3161141	+	-209.4399
71	441.0255	1.4542205	0.035235890	8	0.2312710		0.3740282	-0.6365859				-0.3140566	+	-211.8270
72	441.2982	1.7269444	0.030744257	9	0.2335396	-0.2316226	0.3829028	-0.6431530				-0.3289956	+	-210.7837
88	441.5667	1.9954246	0.026882166	10	0.2612916	-0.2094044	0.3974202	-0.6697541		0.2163355		-0.3252682	+	-209.7154
96	441.7005	2.1292743	0.025141962	11	0.2628133	-0.1516027	0.3771512	-0.5994789	-0.2368850	0.1886733		-0.3578216	+	-208.5561
55	441.9035	2.3322436	0.022715638	9	0.2217168		0.4157906	-0.6078659		0.2749360	0.2834720		+	-211.0864
56	442.7910	3.2197826	0.014574656	10	0.2311636	-0.1818110	0.4180019	-0.5861090		0.2588675	0.2872239		+	-210.3276
63	442.8610	3.2897359	0.014073695	10	0.2182744		0.4027235	-0.5672640	-0.1806598	0.2493479	0.2728322		+	-210.3625
39	442.8625	3.2912014	0.014063386	8	0.1924327		0.3925544	-0.5765301			0.2467928		+	-212.7455
23	443.0857	3.5144134	0.012578244	8	0.2587804		0.3950259	-0.6389480		0.2369378			+	-212.8571
77	443.1312	3.5599653	0.012295001	8	0.2554449			-0.6375345	-0.3116052			-0.3699013	+	-212.8799
47	443.1723	3.6010442	0.012045044	9	0.1915788		0.3795379	-0.5315435	-0.2156434		0.2381680		+	-211.7208
7	443.2127	3.6414671	0.011804040	7	0.2288762		0.3769953	-0.6079200					+	-214.0781
15	443.3375	3.7662223	0.011090227	8	0.2266582		0.3639635	-0.5597670	-0.2252821				+	-212.9830
40	443.3505	3.7792083	0.011018451	9	0.2047991	-0.2040785	0.3966437	-0.5544260			0.2533849		+	-211.8098
109	443.6478	4.0765781	0.009496149	9	0.2264713			-0.6153368	-0.3031703		0.2032495	-0.3642744	+	-211.9585
31	443.7700	4.1987855	0.008933271	9	0.2535168		0.3816314	-0.5934103	-0.1969191	0.2106009			+	-212.0196
8	443.8413	4.2700149	0.008620714	8	0.2414462	-0.1962010	0.3807401	-0.5882033					+	-213.2349

Model	AICc	delta	weight	df	(Intercept)	boldnessF	breathF	chestF	docilityF	escapeF	HRF	NO.callF	sexO	logLik
24	444.0661	4.4947952	0.007704294	9	0.2681758	-0.1762437	0.3970949	-0.6189923		0.2209500			+	-212.1676
64	444.1870	4.6157655	0.007252111	11	0.2272163	-0.1567884	0.4064587	-0.5533899	-0.1551194	0.2390673	0.2777735		+	-209.7994
48	444.2485	4.6772607	0.007032520	10	0.2025835	-0.1719483	0.3846875	-0.5179669	-0.1860471		0.2451523		+	-211.0563
93	444.2729	4.7016623	0.006947239	9	0.2777651			-0.6662483	-0.2890693	0.1670034		-0.3649739	+	-212.2711
125	444.3355	4.7642594	0.006733168	10	0.2491960			-0.6465344	-0.2753047	0.1983413	0.2297988	-0.3576865	+	-211.0998
78	444.4559	4.8846717	0.006339751	9	0.2664795	-0.1531887		-0.6269033	-0.2858257			-0.3704733	+	-212.3626
16	444.5429	4.9716767	0.006069869	9	0.2379772	-0.1619937	0.3684005	-0.5478176	-0.1976511				+	-212.4061
69	444.8702	5.2989416	0.005153647	7	0.2595026			-0.7026255				-0.3174867	+	-214.9068
110	444.8876	5.3163338	0.005109025	10	0.2372060	-0.1613339		-0.6034442	-0.2757658		0.2094833	-0.3647024	+	-211.3758
117	445.1388	5.5675023	0.004506065	9	0.2555044			-0.7075919		0.2360890	0.2455952	-0.3107899	+	-212.7040
101	445.1846	5.6132986	0.004404057	8	0.2286967			-0.6772319			0.2153131	-0.3130260	+	-213.9066
32	445.2041	5.6328369	0.004361242	10	0.2622366	-0.1493675	0.3850595	-0.5820777	-0.1725794	0.2003160			+	-211.5341
85	445.3868	5.8155847	0.003980403	8	0.2864756			-0.7320239		0.2045066		-0.3160944	+	-214.0077
70	445.4540	5.8827472	0.003848956	8	0.2726564	-0.1998622		-0.6838212				-0.3244027	+	-214.0413
102	445.6604	6.0891531	0.003471542	9	0.2415143	-0.2065870		-0.6565245			0.2217295	-0.3199337	+	-212.9648
94	445.7844	6.2131421	0.003262861	10	0.2866278	-0.1420265		-0.6546404	-0.2665469	0.1567767		-0.3658062	+	-211.8242
126	445.7855	6.2142184	0.003261105	11	0.2579436	-0.1488855		-0.6339651	-0.2514379	0.1882172	0.2341956	-0.3584166	+	-210.5986
118	445.9747	6.4034845	0.002966649	10	0.2653189	-0.1873954		-0.6865358		0.2193202	0.2492625	-0.3171527	+	-211.9194
86	446.2897	6.7184473	0.002534387	9	0.2963466	-0.1827388		-0.7124140		0.1877357		-0.3224938	+	-213.2795
13	446.7304	7.1591819	0.002033146	7	0.2537910			-0.6189563	-0.2466380				+	-215.8369
5	446.9607	7.3894559	0.001812029	6	0.2572993			-0.6741793					+	-217.0878

Abbreviations: OF.call, number of calls in the open-field test; NO.call, number of calls in the novel-object test; NO.explo, exploration in the novel-object test; NO, time spent contacting novel objects; HR, heart rate; CORT, cortisol. Suffixes denote individual class: F, ewe; O, offspring.
