# Supplementary material for: Context-Dependent Effects of Maternal Behaviour on Lamb Growth in Tibetan Sheep
Source: Animals (Basel). 2026 May 1;16(9):1386. doi: 10.3390/ani16091386 (PMC13162791; doi:10.3390/ani16091386)
Supplement: Supplementary file 1 [file animals-16-01386-s001.zip › animals-4268266- Table S3.pdf]

Table S3. Candidate model set (AICc) for offspring predictors of offspring growth composite (PC1).

Model	AICc	delta	weight	df	(Intercept)	boldnessO	breathO	CORTO	docilityO	escapeO	explorationO	HRO	NO.exploO	OF.callO	sexO	struggleO	logLik
494	416.9046	0.000000	0.087899797	11	-0.00004831858	-0.4553850		0.2497550	-0.4317135		-0.3458461	0.4344845	-0.4842290	-0.3603993			-196.1582
496	418.1231	1.218466	0.047797080	12	-0.00005290420	-0.4600835	0.1465925	0.2543495	-0.4048258		-0.3346504	0.4242767	-0.4820553	-0.3565085			-195.5170
490	418.1894	1.284789	0.046238045	10	-0.00085832342	-0.4332993			-0.4289478		-0.3423518	0.4277959	-0.4880086	-0.3685263			-198.0267
1006	418.5886	1.683976	0.037871910	12	0.10188882899	-0.4549534		0.2546886	-0.4134435		-0.3305097	0.4272193	-0.4796658	-0.3708199	+		-195.7497
510	418.7276	1.823030	0.035328229	12	-0.00075932977	-0.4406840		0.2408116	-0.4446560	-0.1157105	-0.3306568	0.4497208	-0.4725690	-0.3353714			-195.8193
1518	419.1262	2.221546	0.028945775	12	-0.00004626968	-0.4450050		0.2568356	-0.4319607		-0.3406838	0.4250073	-0.4846883	-0.3660074		0.06948388	-196.0185
492	419.4701	2.565508	0.024372208	11	-0.00867158490	-0.4416318	0.1421732		-0.4145845		-0.3308631	0.4208553	-0.4926395	-0.3677072			-197.4409
506	419.4825	2.577835	0.024222451	11	-0.01694151480	-0.4255464			-0.4647956	-0.1583208	-0.3200815	0.4578176	-0.4825305	-0.3379459			-197.4471
462	419.7570	2.852418	0.021115102	10	-0.00007767185	-0.4088438		0.2461745	-0.4410817			0.3993551	-0.6372008	-0.4082231			-198.8106
512	419.9358	3.031230	0.019309219	13	-0.00089304128	-0.4449271	0.1496686	0.2450290	-0.4178933	-0.1199589	-0.3187045	0.4397677	-0.4699587	-0.3305610			-195.1479
1002	419.9963	3.091726	0.018733893	11	0.09153750767	-0.4324848			-0.4123221		-0.3284418	0.4210876	-0.4839073	-0.3780380	+		-197.7041
1008	420.0289	3.124296	0.018431282	13	0.09047506712	-0.4593805	0.1366132	0.2584193	-0.3904296		-0.3217928	0.4185204	-0.4781504	-0.3660266	+		-195.1945
1520	420.2290	3.324371	0.016676689	13	-0.00004988977	-0.4472928	0.1565666	0.2636065	-0.4033074		-0.3273684	0.4116121	-0.4824872	-0.3633268		0.08776191	-195.2945
1022	420.2809	3.376265	0.016249545	13	0.11251705092	-0.4381714		0.2460959	-0.4250437	-0.1287034	-0.3121228	0.4428888	-0.4656827	-0.3437918	+		-195.3204
1514	420.5421	3.637476	0.014260013	11	-0.00083710379	-0.4266637			-0.4289948		-0.3391656	0.4219434	-0.4883405	-0.3720550		0.04200889	-197.9769
1534	420.5467	3.642115	0.014226975	13	-0.00083737658	-0.4176871		0.2499709	-0.4494658	-0.1573550	-0.3163783	0.4389129	-0.4690305	-0.3358997		0.11881827	-195.4534
464	420.6295	3.724877	0.013650266	11	-0.00008338003	-0.4158714	0.1660585	0.2515085	-0.4102849			0.3890755	-0.6291287	-0.4020647			-198.0206
508	420.6328	3.728172	0.013627794	12	-0.02262699184	-0.4313134	0.1537419		-0.4475711	-0.1711939	-0.3060940	0.4462988	-0.4820932	-0.3325660			-196.7718
458	420.7923	3.887733	0.012582802	9	-0.00744320485	-0.3916933			-0.4464619			0.3967917	-0.6433221	-0.4184919			-200.5308
974	420.9143	4.009681	0.011838503	11	0.13031489858	-0.4109292		0.2526895	-0.4171787			0.3920533	-0.6226879	-0.4188348	+		-198.1630
2030	420.9446	4.039985	0.011660473	13	0.09686855368	-0.4462895		0.2603693	-0.4145508		-0.3269459	0.4196478	-0.4802750	-0.3749989	+	0.05813677	-195.6523
478	421.0436	4.139015	0.011097167	11	-0.00141183485	-0.3923045		0.2344023	-0.4579804	-0.1535385		0.4217741	-0.6127295	-0.3724620			-198.2277
1018	421.2089	4.304311	0.010216889	12	0.09115615198	-0.4222734			-0.4453893	-0.1672969	-0.3044828	0.4511625	-0.4756495	-0.3454669	+		-197.0599
238	421.3987	4.494072	0.009292073	10	-0.00004501989	-0.4617613		0.2600115	-0.3587824		-0.4063723	0.4678479	-0.5163181				-199.6314
474	421.4022	4.497544	0.009275953	10	-0.02561859309	-0.3856464			-0.4857069	-0.2009112		0.4341620	-0.6175358	-0.3746077			-199.6331
1530	421.4303	4.525690	0.009146328	12	-0.02007870320	-0.4088993			-0.4719569	-0.2002767	-0.3053372	0.4498836	-0.4808007	-0.3375182		0.10504818	-197.1706
1536	421.4914	4.586829	0.008870958	14	-0.00104253229	-0.4179003	0.1670471	0.2564590	-0.4206049	-0.1704376	-0.3001976	0.4256772	-0.4654272	-0.3306426		0.14237227	-194.6245
1004	421.5032	4.598627	0.008818786	12	0.07454289749	-0.4393373	0.1321059		-0.3984460		-0.3200425	0.4151138	-0.4871497	-0.3751565	+		-197.2071
1024	421.7394	4.834790	0.007836580	14	0.10031935474	-0.4425599	0.1387979	0.2487772	-0.4029880	-0.1320927	-0.3027867	0.4348769	-0.4642833	-0.3384440	+		-194.7485

Model	AICc	delta	weight	df	(Intercept)	boldnessO	breathO	CORTO	docilityO	escapeO	explorationO	HRO	NO.exploO	OF.callO	sexO	struggleO	logLik
460	421.7609	4.856306	0.007752727	10	-0.01139729947	-0.3998038	0.1616030		-0.4231775			0.3864407	-0.6381321	-0.4145302			-199.8125
1486	421.7669	4.862288	0.007729572	11	-0.00007422955	-0.3964006		0.2553425	-0.4412191			0.3878520	-0.6348582	-0.4145124		0.08928516	-198.5893
1516	421.7880	4.883394	0.007648432	12	-0.00791987731	-0.4328284	0.1480173		-0.4124311		-0.3261139	0.4123070	-0.4925794	-0.3722299		0.05711391	-197.3495
480	421.8782	4.973554	0.007311294	12	-0.00758235105	-0.4012049	0.1706697	0.2329704	-0.4350343	-0.1654424		0.4150348	-0.6050493	-0.3661351			-197.3945
990	422.0016	5.096990	0.006873699	12	0.14201481905	-0.3923562		0.2416553	-0.4320007	-0.1670105		0.4149478	-0.5942250	-0.3803093	+		-197.4562
254	422.0113	5.106727	0.006840315	11	-0.00093549107	-0.4368271		0.2445659	-0.3881329	-0.1899817	-0.3746667	0.4886560	-0.4932511				-198.7116
976	422.0883	5.183639	0.006582260	12	0.11676115594	-0.4171546	0.1522141	0.2569042	-0.3914297			0.3833913	-0.6167961	-0.4120850	+		-197.4996
970	422.1540	5.249425	0.006369271	10	0.11961221463	-0.3891162			-0.4164002			0.3863462	-0.6260652	-0.4258927	+		-200.0091
476	422.1891	5.284436	0.006258743	11	-0.03099517546	-0.3933568	0.1736967		-0.4649257	-0.2130384		0.4213978	-0.6101093	-0.3681828			-198.8004
2046	422.2688	5.364163	0.006014155	14	0.10545226124	-0.4173019		0.2534911	-0.4314226	-0.1671971	-0.2998617	0.4338490	-0.4631480	-0.3437761	+	0.10953987	-195.0132
2032	422.2930	5.388386	0.005941755	14	0.08304424660	-0.4482421	0.1461633	0.2661871	-0.3902832		-0.3164745	0.4079076	-0.4788492	-0.3712134	+	0.07681915	-195.0253
1532	422.3408	5.436186	0.005801431	13	-0.02635302680	-0.4114826	0.1710607		-0.4535728	-0.2255115	-0.2865793	0.4354174	-0.4799109	-0.3323143		0.13111270	-196.3504
1502	422.3458	5.441174	0.005786980	12	-0.01011873714	-0.3703685		0.2391150	-0.4729593	-0.2184687		0.4165467	-0.6016960	-0.3717757		0.15679219	-197.6283
2026	422.4423	5.537704	0.005514305	12	0.08873971528	-0.4275714			-0.4128656		-0.3264943	0.4169374	-0.4842796	-0.3803747	+	0.03125617	-197.6766
1488	422.4671	5.562484	0.005446402	12	-0.00007817805	-0.4011589	0.1779389	0.2631024	-0.4082473			0.3742741	-0.6256860	-0.4093137		0.10917764	-197.6890
240	422.4940	5.589352	0.005373726	11	-0.00004664397	-0.4666682	0.1553808	0.2647688	-0.3311121		-0.3938124	0.4566461	-0.5136443				-198.9529
250	422.6170	5.712422	0.005053022	10	-0.02511150307	-0.4281759			-0.4204263	-0.2394745	-0.3636075	0.4951273	-0.5064626				-200.2406
1020	422.6179	5.713328	0.005050733	13	0.07396885463	-0.4276444	0.1420562		-0.4304967	-0.1769629	-0.2942589	0.4423545	-0.4759944	-0.3401993	+		-196.4890
986	422.6608	5.756162	0.004943713	11	0.11472012874	-0.3825553			-0.4581797	-0.2077045		0.4268782	-0.6005370	-0.3824786	+		-199.0363
1504	422.7471	5.842524	0.004734782	13	-0.01677862019	-0.3766069	0.1945971	0.2397063	-0.4486235	-0.2435635		0.4045705	-0.5905180	-0.3638191		0.18617240	-196.5536
234	422.8124	5.907831	0.004582670	9	-0.00083678735	-0.4388975			-0.3542584		-0.4042416	0.4614308	-0.5209801				-201.5408

Abbreviations: OF.call, number of calls in the open-field test; NO.call, number of calls in the novel-object test; NO.explo, exploration in the novel-object test; NO, time spent contacting novel objects; HR, heart rate; CORT, cortisol. Suffixes denote individual class: F, ewe; O, offspring.
